# Correspondence

**Published:** 1890-02

**Authors:** C. D. Roy

**Affiliations:** Charity Hospital, New York


NEW YORK, January 4, 1890.

If I may hope that the rambling subjects spoken of in my letters may prove themselves of benefit, and I trust of interest to the readers of THE RECORD, I shall take pleasure in interesting their thoughtful reveries by thrusting before their eyes some facts and scenes from the metropolitan city. That scenes and occurrences in New York are far more interesting to the observing eye than can ever be depicted in written language, is obvious to writers of all departments of literature, *a fortiori*

does it hold true in the realm of medicine. To the laity, the word doctor has a hallowed ring, a something so mysteriously strange, and yet he is often hallowed only by those who lie upon a couch of sickness.

A physician's life in this city for the first few years of his fœtal existence is not one of envy. The city itself is flooded with practitioners of medicine, and nowhere does a young man have to undergo the same drudgery in order to get a foothold that he does in this place. This, of course, applies not to the young physicians with "pull," but to those who have to rise by the power of their brains. It is a place where specialists hold chief sway, and where their existence is attended with marked success. It was my pleasure to be present a few nights ago at the meeting of the Academy of Medicine. I knew in advance that the discussion would be of interest from the fact that it was to be participated in by such noted men as Drs. Loomis, Janeway, J. Lewis Smith and others. Before remarking upon the discussion which occurred, I shall pause to speak of the Academy, in hopes of giving some information to those who may sometime wish to attend its meetings, and points which it is oft-times difficult to obtain. The building now used for the Academy is one situated in the row of structures on the south side of Thirty-first street, between Fifth avenue and Broadway. Their meetings, which are always interesting and profitable, since they are conducted by those most prominent in the medical profession, occur on the last Monday night in each month. These meetings are free and open to all, and will prove very interesting to any member of the profession who may visit New York. A new and elegant building, on Forty-third street, just off of Fifth avenue, is now in course of erection, which is to be owned and used exclusively by the Academy of Medicine. It will perhaps be ready for occupancy by the first of next May, and when completed will be a building of imposing structure. The paper read upon the night referred to was by Dr. Chapin upon "The Existence of a Pre-systolic Murmur in Certain Conditions of Children." He held that there often existed this murmur in children who had never suffered with rheumatism, but apparently due to some congenital formation of the heart, but which disappeared with advancing years. He quoted numerous cases where this condition was found, and where no cause could be given for the seeming mitral stenosis, and where hæmic murmurs frequently existed. In the discussion which followed Dr. Loomis said he thought there was no doubt at present in the minds of physicians that there was such a thing as pre-systolic murmur, confirming in his own experience the views expressed by Dr. Chapin. Following Dr. Lewis, Dr. Janeway spoke upon the subject in question. Dr. J. was listened to with great attention, and is considered here one of the brightest men in the profession. He said that in teaching he was accustomed to class the so-called pre murmurs with the systolic, for clinically he did not believe the distinction could be made. He said that there might be a pre first sound he admitted, but whether this was before the actual systolic murmur he could not say, for no one can tell the exact time of the commencement of a systolic act. Dr. J. Lewis Smith also gave to his auditors the expression of his views on the subject, which were indeed valuable as coming from one of such vast clinical experience with children.

Before closing my letter I shall barely make mention of a rare occurrence which was seen at Vanderbilt clinic. It is the only case I know of its kind in which heredity played such a prominent part. Three sisters presented themselves at the gynæcological clinic to be treated for uterine complaints. On examination the following condition of affairs presented themselves: The first was found to have a unicorn uterus; the second had one uterus with two cervices, while the last had no uterus or vagina, but simply a small vulvo-vaginal opening, where the meatus urinarius opened. The cases were unique, and for that reason I thought it would be of interest to some of the readers to know of such an occurrence.

At present the various colleges are closed for the Christmas vacation, although the weather presents none of those signs of a "freezing blizzard." C. D. ROY, M. D.

*Charity Hospital, New York.*

---

PREVENTION OF MAMMARY ABSCESS.—Dr. Altamirano recommends massage and aspiration combined to empty the female breast in order to prevent mammary abscess.—*Gaceta Medica,* City of Mexico.

